# Intestinal Obstruction due to Bilateral Strangulated Femoral Hernias

**DOI:** 10.1155/2014/195736

**Published:** 2014-06-26

**Authors:** Ioannis Nikolopoulos, Eshan Oderuth, Eleni Ntakomyti, Bengt Kald

**Affiliations:** ^1^General Surgery, Lewisham and Greenwich NHS Trust, London SE18 6SY, UK; ^2^General and Breast Surgery, Lewisham and Greenwich NHS Trust, London SE18 4QH, UK

## Abstract

*Introduction*. Femoral hernias are at high risk of strangulation due to the narrow femoral canal and femoral ring. This can lead to symptoms of obstruction or strangulation requiring emergency surgery and possible bowel resection. To our knowledge, there is only one previous published report of bilateral strangulated femoral hernia. We present our case of this phenomenon. *Case Report*. An 86-year-old woman presented with symptoms of small bowel obstruction. Examination revealed two tender lumps in the area of the femoral triangle. CT scan revealed bilateral femoral hernias. Both hernias were repaired and a small bowel resection on the right side was performed with side to side anastomosis. She made an uneventful recovery. *Conclusion*. Bilateral femoral hernias are a rare occurrence with only one reported case of bilateral strangulation. Our case highlights the importance of meticulous history taking and clinical examination as any delay in diagnosis will increase the risk of mortality and morbidity for the patient. Hernias should always be considered as a cause if one presents with symptoms of abdominal pain or obstruction.

## 1. Introduction

A femoral hernia is a protrusion of a viscous through the femoral canal due to a defect in the femoral ring. It is the third commonest hernia with 20% occurring in females compared to 5% in men. This hernia is more prevalent in parous elderly women and found on the right side. Femoral hernias descend through the femoral ring then femoral canal and exit via the saphenous opening. The femoral ring is bounded anteriorly by the inguinal ligament, posteriorly by the iliopectineal ligament, medially by the lacunar ligament, and laterally by the femoral vein. They are the most likely to become strangulated due to the narrow femoral canal and rigid femoral ring. Strangulation may lead to bowel resection which has been shown to have increased mortality and morbidity [1]. The aetiology of femoral hernias is a controversial issue due to lack of evidence in terms of congenital versus acquired theory. The acquired theory is widely accepted with a general explanation of increased intra-abdominal pressure from bronchitis, constipation, and pregnancy leading to stretching of the femoral ring from a dilated femoral vein [2, 3].

Clinical features of a femoral hernia may be the sensation of a lump in the groin but may cause a dragging sensation. Symptoms of colicky pain may persist due to incarceration and develop with vomiting due to obstruction or strangulation of small bowel. On examination the hernia can be identified below and lateral to the pubic tubercle; it may be generally irreducible and may be tender [2, 4]. A femoral hernia needs to be distinguished clinically from other groin lumps such as inguinal hernia, saphena varix, enlarged femoral lymphadenopathy, lipoma, femoral aneurysm, and psoas abscess. Generally diagnosis is by clinical means; however, imaging techniques using ultrasound, CT, or MRI may be used. If diagnosis is still a doubt then diagnostic laparoscopy is an option [5]. Treatment consists of swift operative management to repair the defect. There are three open approaches to repair using either the Lockwood (low), McEvedy (high), or Lotheissen's (transinguinal) operation. Laparoscopic approaches include the totally extraperitoneal approach (TEP) or the transabdominal preperitonoeal approach (TAPP). Our case provides evidence for the very rare occurrence of bilateral strangulated femoral hernia. There has been one published case report since 1942 of this phenomenon [6]. We also highlight the need to examine for hernias in patients presenting with abdominal pain and signs of obstruction.

## 2. Case Report

An eighty-six-year-old woman from a nursing home presented to the Accident and Emergency Department with absolute constipation for three days, constant abdominal pain, distension, and vomiting. It was also noted that the patient had lost twenty-five kilograms in weight in approximately three to four months and reduced appetite. Her past medical history included a sliding hiatus hernia, diverticular disease, dementia and multiple deep vein thrombosis, and pulmonary embolism for which she was on warfarin.

On examination the patient appeared comfortable at rest. She had abdominal distension and tenderness particularly in the lower half of the abdomen with no evidence of peritonism, no palpable masses, and no organomegaly. Examination of the groin revealed two well circumscribed lumps in the region of the femoral triangle. They were tender to palpation. There was no change in the colour or temperature of the overlying skin. There were no audible bowel sounds or bruit over the lumps.

Blood tests showed neutrophils 21 10^9^/L, white cell count 23 10^9^/L, creatinine 65 *μ*mol/L, complement reactive protein 45 mg/L, lactate 3.2, INR 2.7, sodium 127 mmol/L, potassium 4.4 mmol/L, urea 7.7 mmol/L, serum glucose 4.5 mmol/L, amylase 29 *μ*/L, bilirubin 4 *μ*mol/L, albumin 18 g/L, alkaline phosphatase 320 *μ*/L, alanine transaminase 19 *μ*/L, adjusted calcium 2.38 mmol/L, haemoglobin 109 g/L, and platelets 285 10^9^/L.

**Figure 1:**
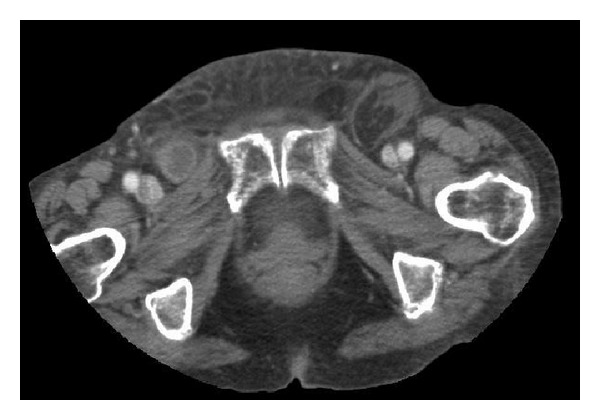
CT abdomen and pelvis transverse view with IV contrast illustrating bilateral femoral hernia containing small bowel.

**Figure 2:**
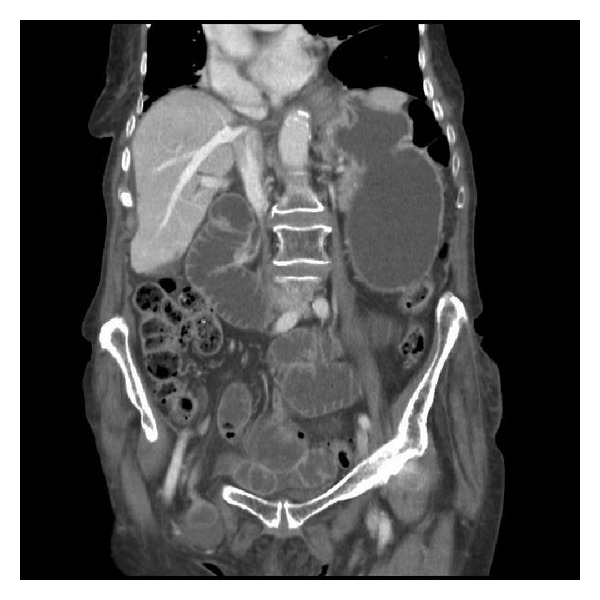
CT abdomen and pelvis coronal view with IV contrast illustrating right sided femoral hernia containing small bowel and small bowel obstruction.

**Figure 3:**
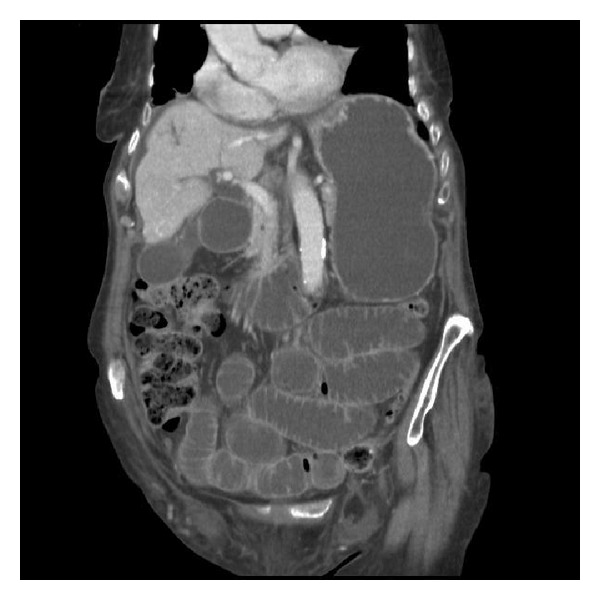
CT abdomen and pelvis coronal view with IV contrast illustrating left sided femoral hernia containing small bowel and small bowel obstruction.

In view of this patient's significant weight loss a CT abdomen and pelvis was organised to exclude the possibility of malignancy contributing to the clinical picture of bowel obstruction (Figures 1, 2, and 3). The CT reported the presence of bilateral groin hernias and the patient underwent an emergency operation six hours after she was initially assessed by the duty surgical team. In order to normalise the INR prior to surgery, she received two units of fresh frozen plasma on the advice of the Haematology team and the INR before operating had come down to 1.5.

McEvedy's approach was preferred bilaterally in order to gain access to the peritoneal cavity if necessary. There was a small knuckle of ischaemic gangrenous bowel strangulated at the right femoral hernia. Left femoral hernia contained a small loop of strangulated small bowel which was still viable. The ischaemic bowel was resected and a side to side anastomosis was performed using a linear stapler device. Prolene interrupted sutures were used to close the hernia defects. The patient was admitted to intensive care postoperatively and discharged to the ward after 2 days. She made a full recovery and was discharged home within one week.

## 3. Discussion

Bilateral femoral hernias are very rare occurrence. We have found just over a dozen reported cases of bilateral femoral hernia in the literature with only one reported case of bilateral strangulated hernia [6, 7].

The operation was performed through bilateral McEvedy's approach using a small oblique incision 3 cm above the pubic tubercle extending to the lateral border of the rectus muscle. This was used due to the emergency nature of the operation so that the abdominal cavity could be accessed, which proved useful for resecting part of the small bowel from the right sided femoral hernia. The defect was repaired with interrupted prolene sutures. Although the operation was delayed for about six hours in order to correct the INR, we feel that this was probably not responsible for the state of small bowel ischaemia encountered during the procedure as the patient presented late with symptoms and signs of bowel obstruction for three days.

In our experience, high McEvedy's approach is generally preferred in the emergency setting as this provides good access to the peritoneal cavity allowing easier evaluation of the small and large bowel and facilitates adequate resection if necessary [8]. Repair can be performed with either primary sutures or mesh repair. Mesh repair is used in a higher proportion of elective than emergency cases. The reason for this is that in emergency cases often bowel or omentum is resected due to strangulation which is an environment which would increase the risk of mesh infection, if peritonitis or an abscess especially is apparent [2]. Mesh repair has been shown to have a reduced recurrence rate compared with primary suture repair in the elective setting with no significant differences found in the emergency setting [1, 5]. Increased recurrence has been found in femoral hernias compared to inguinal hernias and emergency compared to elective cases [1].

Laparoscopic approaches have been shown to have lower recurrence rate and postoperative pain compared to open techniques. However laparoscopy requires more time, specialised skill and is more expensive [9].

## 4. Conclusion

In conclusion, this case report of a rare phenomenon of bilateral strangulated femoral hernias reinforces the importance of femoral hernias due to their high risk of strangulation. One should be vigilant in patients presenting with gastrointestinal symptoms, especially in case of suggestive of small or large bowel obstruction. Elderly frail patients especially with obstructed femoral hernias may present with atypical symptoms of abdominal pain, nausea, and vomiting. Therefore, meticulous clinical examination including thorough examination of both inguinal areas, complemented by appropriate haematological and radiological investigations, is essential in the diagnosis of these hernias. Any delay or failure to reach this diagnosis would result in a significantly increased risk of morbidity and mortality for the patient.
